# A rare case of numerous hydatid cysts in the pleural cavity without extrapleural involvement

**DOI:** 10.1016/j.amsu.2021.102290

**Published:** 2021-04-13

**Authors:** Albaraa Bara, MHD Othman AlKhatib, Issam Alkhayer

**Affiliations:** a Faculty of Medicine, Damascus University, Damascus, Syrian Arab Republic; b Department of Thoracic Surgery, Al-moassat Hospital, Damascus, Syrian Arab Republic

**Keywords:** Case report, Hydatid cyst, Pleural hydatidosis, Hemithorax, Pleura

## Abstract

**Introduction:**

and importance: The Echinococcus Granulosus, an endemic parasite in several parts of the world which causes hydatid disease. Human acts as an intermediate host and gets infected by eating parasitic eggs. As it is well known, lungs and liver are the most commonly involved organs in this disease. When the pleura is involved, it is almost always secondary to a ruptured primary lung cyst.

**Case presentation:**

The purpose of this paper is to present a case of 16-year-old male with complaints of dyspnoea and dry cough for 6 months. His vital signs, CBC, and laboratory tests were all within normal. Chest X-ray showed a complete opacification of hemithorax.

**Clinical discussion:**

CT revealed multiple cysts filling up the whole pleural cavity with collapsed lung and to-left mediastinal shift. The patient was diagnosed with primary pleural hydatidosis. The treatment was surgical, followed by parasitic medications. During the surgery, surgeons were able to simply extract many cysts by hand and eventually the collapsed lung returns to its normal volume and normal functional state. The patient was indicated to continue with Albendazole for 1 year after surgery. Three days after the surgery, chest X-ray was within normal.

**Conclusion:**

Primary pleural hydatidosis is such a rare case to present as full filled up pleural cavity and a complete opacification of hemithorax on CXR. Using the developed technical methods helped to confirm such case and in choosing the best surgical intervention. The result was satisfactory with fully expanded and functional lung.

## Highlights

• Primary pleural hydatidosis is an extremely rare clinical entity.
• Liver and lungs are the most common locations for primary cysts.
• Hydatid disease could be asymptomatic or present with nonspecific symptoms.
• Surgery is the main treatment for hydatid disease, followed by oral medications.
• Prognosis is excellent in hydatid disease after surgery.

## 1 Introduction

Hydatid disease is an anthropozoonosis caused by Echinococcus granulosus, it is still endemic in several parts of the world especially in developing countries [1]. The definitive hosts for E.granulosus are Carnivores, Human accidently acts as an intermediate host when he gets infected by eating contaminated food or water containing the parasitic eggs, or when he is directly contacted to the definitive hosts (dogs, wolfs, foxes) [2].

As it is well known, hydatid cysts could exist in multiple locations in the body, but Liver represents the most commonly involved location, while Lungs are the most common site for the extrahepatic disease [3]. To the best of our knowledge, the pleural cavity is an extremely unusual location for primary hydatid cysts, and the majority of reported pleural hydatidosis cases are secondary to a ruptured primary cyst in the peripheral lung [2]. The usual progress of the pleural disease is to be a solitary cyst, and the main treatment is surgical [3].

Here in our case, we had numerous of small cysts showing up on chest X-ray as a complete opacification of hemithorax, and the surgeon was able to simply extract many of them by hand. This work has been reported in line with the SCARE criteria [4].

## 2 Case presentation

**Fig. 1:**
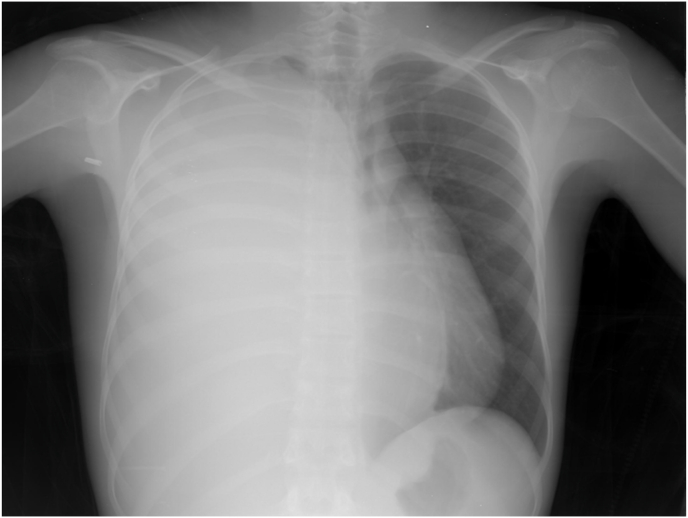
Patient's chest X-ray: a complete opacification of hemithorax on the right side.

A 16-year-old non-smoker and non-drinker male presented to our clinic complaining of dyspnoea, and dry cough that started 6 months before and exacerbated during the last 2 months, he had a history of falling from 2 m height occurred 3 years ago. On physical examination, the patient was hemodynamically stable, his blood pressure, heart rate, temperature, and respiratory rate at rest were all within the normal ranges, breathing sounds were absent in the right side, also vocal fremitus were found to be absent, along with dullness on percussion over the right side of the chest, clinical examination of the left side was normal, his laboratory tests were within the normal ranges. Chest X-ray revealed a complete opacification of hemithorax on the right side (Fig. 1).

**Fig. 2:**
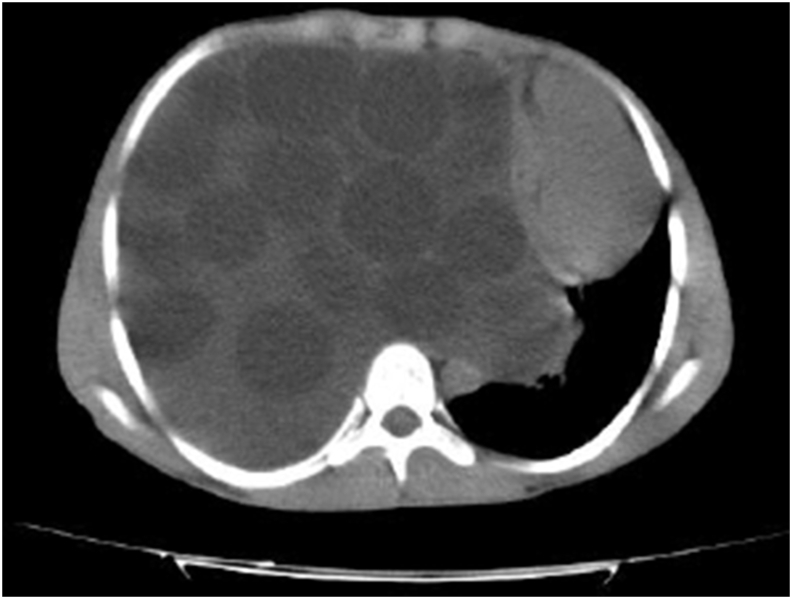
Patient's CT scan: multiple round-shaped cystic formations filling up the entire right pleural cavity.

Computed tomography revealed multiple round-shaped cystic formations filling up the entire right pleural cavity along with collapsed right lung and to-left mediastinal shifting (Fig. 2).

**Fig. 3:**
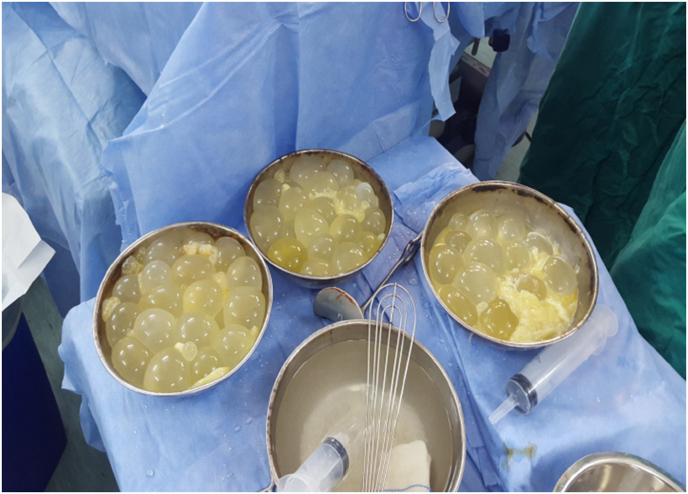
Hydatid cysts after extraction from pleural cavity.

The patient then underwent surgery by our well experienced surgical team in Al-moassat hospital. We did a right posterolateral thoracotomy through the fifth intercostal space, the parietal pleura was irregularly thickened. Hypertonic saline was introduced into the pleural cavity to kill the scolices, then the pleural cavity was opened. The pleural cavity was discovered after draining 2 l litres of yellow coloured fluid, it was filled up with too many small secondary hydatid cysts, we also found some ruptured cysts, as well as large laminated layer which may be belonged to the ruptured primary cyst. The laminated layer was removed, as well as hundreds of secondary hydatid cysts (Fig. 3), which were removed easily by hand **(video. 1)**, the pleural cavity was completely evacuated then repeatedly washed, the lung was trapped. After surgical decortication, the collapsed right lung was fully re-expanded, there was no parenchymal damage.

**Fig. 4:**
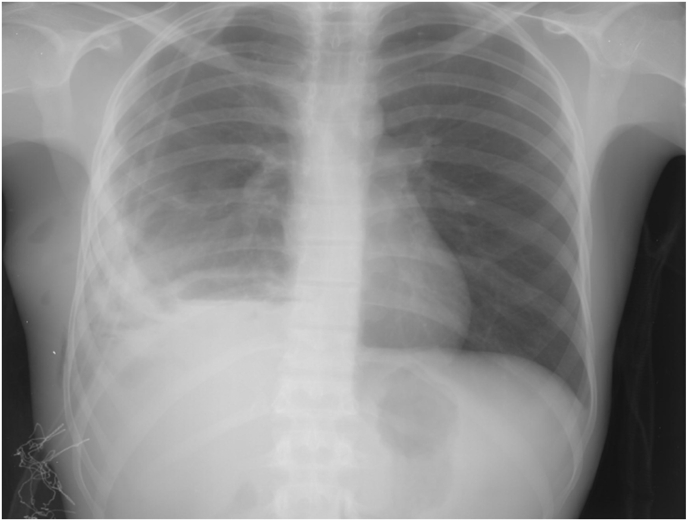
Patient's chest X-ray 3 days postoperatively, a fully re-expanded right lung.

The patient was extubated immediately after the surgery and he had uneventful recovery. As a postoperative follow up, daily chest X-ray was performed and the lung was fully re-expanded 3 days after the surgery (Fig. 4). The patient was discharged from the hospital after 5 days with the indication of continuing his medical treatment with 10 mg/kg/day of oral Albendazole for 1 year. Chest X-ray in a follow up visit after 1 month was within normal.

Supplementary video related to this article can be found at https://doi.org/10.1016/j.amsu.2021.102290

The following is the supplementary data related to this article:

Video

## 3 Discussion

Hydatid cysts can develop in multiple locations in the body, Intrathoracic but extrapulmonary disease; involving the presence of hydatid cyst in the pleura, heart, pericardium, mediastinum, chest wall, and diaphragm, is an extremely rare clinical entity which represents a diagnostic challenge, especially in cases without a primary cyst in a common location [5]. Most primary hydatid cysts develop in the lung or in the liver, while secondary cysts could be found anywhere due to the possibility of haematogenous dissemination [6]. The most common mechanism of pleural hydatidosis is the formation of the cysts secondary to a primary one, as concluded in the first important study on secondary pleural hydatidosis in 1937 by Dévé, who proclaimed that primary pleural hydatidosis in not favorable as a mechanism [7]. However, latter studies accepted the presence of this pathology [8].

Primary pleural hydatidosis is defined by the presence of a primary hydatid cyst in the pleural cavity, or a parasitic pleural effusion. According to Erkoc, M. et al., less than 1% of all hydatidosis cases are primary pleural hydatidosis [9].

Hydatid cyst can develop in the pleural layers, due to the permeability of the laminated layer to water, urea, calcium, chloride, and other nutrients that can pass through the membrane by diffusion [10].

In our case, the cysts were found only in the pleura, we removed the laminated membrane of the primary cyst along with many small secondary cysts, the parasitic pleural effusion was also drained. We could not find any extrapleural lesions, and then we concluded that the diagnosis was primary pleural hydatidosis, and this represents an extremely rare clinical entity.

Pleural hydatidosis can mimic the clinical presentation of pleural effusions such as dyspnoea, chest pain, dry cough, and to-shift mediastinal shift, as it can be asymptomatic in 15% of cases [11].

The diagnosis of pleural hydatidosis is mainly based on the radiological imaging. In most cases, chest X-ray and CT scan are able to confirm the diagnosis and help in the planning for surgery, which is the main treatment for hydatid disease in addition to the postoperative antiparasitic therapy [12].

## 4 Conclusion

Primary pleural hydatidosis is a very rare disease with very few reported cases. In our case, it was confirmed depending on series of medical diagnostic methods, starting with the chief complaint and clinical story to CT scan. The prognosis was very good, the lung re-expanded to full size and functional ability. It is very important to do CXR on follow up to detect any recurrence, it is also essential to indicate anti-parasitic medications to keep recurrence rate low.

## Informed consent

Written informed consent was obtained from the patient for publication of this case report and accompanying images. A copy of the written consent is available for review by the Editor-in-Chief of this journal on request.

## Provenance and peer review

Not commissioned, externally peer-reviewed.

## Ethical approval

None.

## Sources of funding

None.

## Author contribution

MA: reviewed the literature, wrote the abstract, and the introduction.

AB: reviewed the literature, wrote the case presentation, and the discussion.

IA; lead the surgical team, checked the spelling and grammar, revised the manuscript and helped in writing the discussion.

## Research registration

None.

## Registration of research studies

N/A.

## Guarantor

Mr. MHD Othman AlKhatib

## Consent

Written informed consent was obtained from the patient for publication of this case report and accompanying images. A copy of the written consent is available for review by the Editor-in-Chief of this journal on request.

## Declaration of competing interest

All of the authors declared that they have no conflict of interest.
